# Supplementary material for: Comparative Transcriptome Analysis Reveals Regulatory Networks during the Maize Ear Shank Elongation Process
Source: Int J Mol Sci. 2021 Jun 29;22(13):7029. doi: 10.3390/ijms22137029 (PMC8268914; doi:10.3390/ijms22137029)
Supplement: Supplementary file 1 [file ijms-22-07029-s001.zip › Figure S1 and Table S1.pdf]

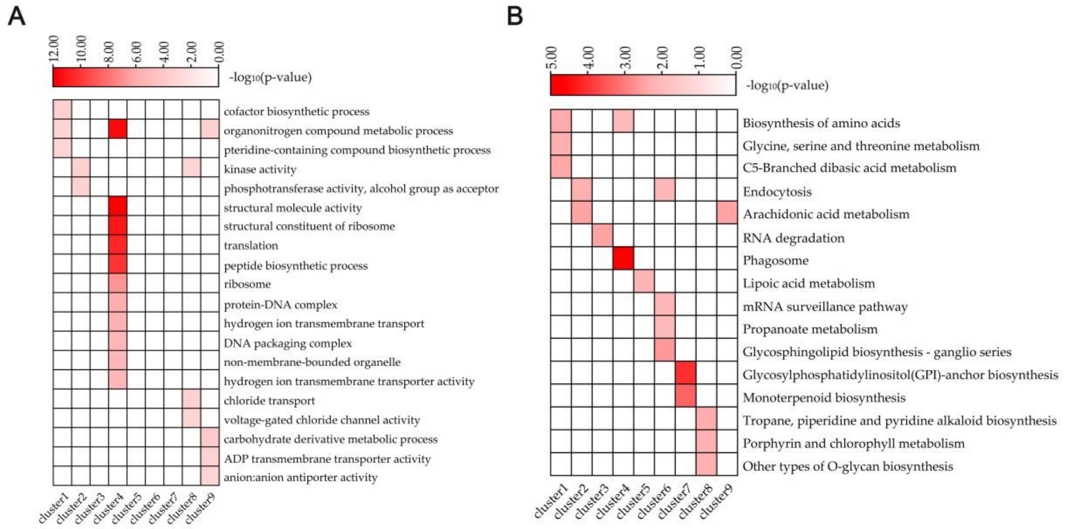

Figure S1: Significant categories of GO and KEGG in different clusters. A. The GO terms within the different clusters. Only significant categories (*p*-value< 0.001) were displayed. B. KEGG pathways within the different clusters. Only significant categories (*p*-value< 0.001) were displayed.

Table S1. Overview of the RNA-seq data.

Sample	Raw Reads(M)	Clean Reads(M)	Q30 (%)	Mapped Reads (M)(%)	Unique Mapped Reads (M)(%)	Multiple Mapped Reads (M)(%)
L1-1	23.27	22.56	93.81	20.35(90.99)	19.78(88.51)	0.57(2.48)
L1-2	25.43	24.71	94.51	23.08(91.38)	22.45(88.88)	0.63(2.50)
L2-1	23.85	23.07	94.15	21.04(91.12)	20.44(88.53)	0.60(2.59)
L2-2	23.2	22.75	93.69	20.85(87.83)	20.25(85.31)	0.60(2.52)
L3-1	22.9	21.25	94.66	18.32(82.01)	17.80(79.48)	0.53(2.53)
L3-2	20.07	19.55	94.82	18.33(91.91)	17.80(89.27)	0.53(2.64)
L4-1	24.03	23.37	94.75	21.96(92.00)	21.27(89.14)	0.68(2.86)
L4-2	24.04	23.41	94.77	21.92(91.77)	21.21(88.81)	0.71(2.96)

Notes: L1, L2, L3, and L4 stand for four lengths of the second internodes (1 cm, 2 cm, 3 cm, 4 cm) during the shank elongation. -1and -2 represent the two biological replicates per length. M represents million. Q30 represents the percentage of nucleotides with a quality value≥30.
